# Aquatic microbial diversity associated with faecal pollution of Norwegian waterbodies characterized by 16S rRNA gene amplicon deep sequencing

**DOI:** 10.1111/1751-7915.13461

**Published:** 2019-07-09

**Authors:** Lisa Paruch, Adam M. Paruch, Hans Geir Eiken, Roald Sørheim

**Affiliations:** ^1^ Division of Environment and Natural Resources Norwegian Institute of Bioeconomy Research (NIBIO) Fredrik A. Dahls vei 20 1433 Aas Norway

## Abstract

Faecal contamination is one of the major factors affecting biological water quality. In this study, we investigated microbial taxonomic diversity of faecally polluted lotic ecosystems in Norway. These ecosystems comprise tributaries of drinking water reservoirs with moderate and high faecal contamination levels, an urban creek exposed to extremely high faecal pollution and a rural creek that was the least faecally polluted. The faecal water contamination had both anthropogenic and zoogenic origins identified through quantitative microbial source tracking applying host‐specific *Bacteroidales* 16S rRNA genetic markers. The microbial community composition revealed that *Proteobacteria* and *Bacteroidetes* (70–90% relative abundance) were the most dominant bacterial phyla, followed by *Firmicutes*, especially in waters exposed to anthropogenic faecal contamination. The core archaeal community consisted of *Parvarchaeota* (mainly in the tributaries of drinking water reservoirs) and *Crenarchaeota* (in the rural creek). The aquatic microbial diversity was substantially reduced in water with severe faecal contamination. In addition, the community compositions diverge between waters with dominant anthropogenic or zoogenic pollution origins. These findings present novel interpretations of the effect of anthropo‐zoogenic faecal water contamination on microbial diversity in lotic ecosystems.

## Introduction

Faecal water contamination originates from anthropogenic and zoogenic sources associated with both point (typically, sewage and wastewater discharges) and nonpoint/diffuse (mostly storm/urban/agricultural water runoff) pollution. In addition, the contamination occurs in groundwater and surface waterbodies through direct (faeces and droppings) and indirect (organic fertilization, drainage and irrigation of soil and vegetation) pathways. These are the main transmission routes to water environments for various microbiota species derived from faecal pollution (Paruch and Paruch, 2018).

The multiple sources and origins of faecal water contamination create substantial challenges for engineers and scientists in applying adequate measures for managing and treating polluted waters, assessing water quality status and protecting aquatic environments. In response to these, microbial source tracking (MST) methods using quantitative polymerase chain reaction (qPCR) have been widely implemented into practice. The qPCR‐based MST techniques detect and quantify host‐specific genetic markers, which determine the origins of faecal pollution. The most tested, validated and applied markers in aquatic research are those derived from *Bacteroidales* 16S rRNA genes (Hagedorn *et al.*, 2011; Reischer *et al.*, 2013; Harwood *et al.*, 2014; Staley *et al.*, 2016; Paruch *et al.*, 2017; Sowah *et al.*, 2017).

Surface water environments are more vulnerable to contamination than groundwater, and their microbial profiles represent the most important indicators of biological water quality. It is therefore highly important to assess faecal contamination impact (with particular emphasis on anthropogenic and zoogenic origins) on the profile of aquatic microbiota in the natural water ecosystems. In this regard, next‐generation sequencing (NGS), for example Illumina MiSeq, has been successfully employed for the characterization of microbial community structure (Wang *et al.*, 2016; Calderón *et al.*, 2017; Parulekar *et al.*, 2017). Yet, the composition of aquatic microbiota impacted by faecal pollution remains sparsely addressed. More specifically, there are no available published data focusing on the impacts of both anthropogenic and zoogenic faecal sources on microbial population in natural waters. The most relevant studies investigated bacterial diversity in either lentic waters or benthic microbiota in marine ecosystems exposed only to anthropogenic pollution (Zinger *et al.*, 2012; Zeglin, 2015; Ibekwe *et al.*, 2016; Calderón *et al.*, 2017; Parulekar *et al.*, 2017). Therefore, more comprehensive studies, which can provide a better overview of the diversity of various microbial communities impacted by faecal pollution in lotic environments, are needed (Zinger *et al.*, 2012).

In this study, we focused on investigating microbial community changes under the pressure of anthropo‐zoogenic faecal water contamination in different lotic ecosystems. The core research question we set out to address was the following: does faecal water pollution increase or decrease the aquatic microbial diversity in lotic environments?

## Results and discussion

### Origins of faecal contamination in lotic waters

Various lotic ecosystems (comprising a rural creek – RC, an urban creek – UC and three tributaries of different drinking water reservoirs – T1, T2 and T3) were investigated, and all of them revealed constant exposure to faecal pollution based on *Escherichia coli* (*E. coli*) concentrations. Therefore, our main focus was given to the most distinctive samples exposing extreme variation in *E. coli* counts (lowest vs. highest) and those with divergent origins of faecal pollution. The origin (anthropogenic and zoogenic/environmental) was defined through quantitative microbial source tracking (QMST) using host‐specific *Bacteroidales* 16S rRNA genetic markers (AllBac – universal marker, BacH – human, BacR – ruminant and Hor‐Bac – horse) and was further presented as the percentage contribution profile of the markers in faecal pollution. The methodology of contribution profiling and its scientific background have been described in greater detail elsewhere (Paruch *et al.*, 2015). Although there are no significant correlations between *E. coli* and the *Bacteroidales* genetic markers (Harwood *et al.*, 2014), molecular diagnostics using these markers are quite prevalent in the identification of the origin of faecal water contamination. *Bacteroidales* bacteria, especially species of the genus *Bacteroides*, constitute the majority of the population of gastrointestinal microbes. Normally, these species comprise one third of total faecal bacteria, but they can extend to over half the portion of human faecal flora, constituting 10^11^ organisms in a gram of faeces (McQuaig *et al.*, 2012).

The lowest *E. coli* concentrations were found in the rural creek (RC), where the contribution profile of the tested *Bacteroidales* DNA markers showed a predominantly zoogenic origin (Table 1). A converse profile was determined in the urban creek (UC), which had faecal contamination of a predominantly anthropogenic origin and extremely high *E. coli* numbers. Relatively high *E. coli* concentrations were also found in the tributaries of drinking water reservoirs (T1, T2 and T3), where the contribution profile of genetic markers in faecal water contamination exposed a dominant zoogenic origin (Table 1).

**Table 1:** The lowest and highest concentrations of *Escherichia coli* (*E. coli*) expressed as the most probable number (MPN) in 100 ml of water sampled from various lotic ecosystems (a rural creek – RC, an urban creek – UC and three tributaries of different drinking water reservoirs – T1, T2 and T3)

Study sites	*E. coli* concentrations	Number of samples per dominant origin of faecal water contamination		Total number of tested samples
		Anthropogenic	Zoogenic	
RC	1–144.5	1	9	10
UC	560–20 050	8	2	10
T1	1–200.5	2	13	15
T2	2–200.5	0	10	10
T3	2–200.5	2	13	15

### Microbial taxonomic diversity of faecally polluted lotic ecosystems

The microbial diversity of the various lotic ecosystems was investigated using Illumina MiSeq sequencing on 16S rRNA amplicon libraries. A range of 1 267 757 to 1 850 305 reads were obtained per single sample investigated. After quality filtering and paired‐end sequence assembly, 615 567 unique reads were used to characterize 49 107 operational taxonomic units (OTUs). These OTUs were further classified into 74 microbial phyla, with a majority of bacteria (71) and three archaeal phyla.

*Proteobacteria* and *Bacteroidetes* were the dominant populations (constituting 70–90% relative abundance) in the investigated lotic ecosystems. In addition, the typical human gastrointestinal microbiota represented by *Firmicutes* (Goodrich *et al.*, 2014; Johnson *et al.*, 2017) was highly distinctive in the UC ecosystem. Over 50% of this phylum was represented by the genus *Faecalibacterium*, an essential bacterium in the human gut (Miquel *et al.*, 2013). This specific finding clearly indicates the impact of human faecal contamination on aquatic microbial diversity, and it is in agreement with the results from QMST, demonstrating the dominant anthropogenic origin of faecal pollution in the UC ecosystem (Table 1). Such a relationship was recently reported by Sun *et al*. (2017), revealing an anthropogenic source (wastewater discharge) of *Firmicutes* in the Yangtze River.

*Parvarchaeota* and *Crenarchaeota* were the main archaeal population of the investigated ecosystems. The exclusive predominance (up to 86% relative abundance) of *Parvarchaeota* was revealed in all three tributaries (T1, T2 and T3), while *Crenarchaeota* constituted the second largest phylum, representing up to 44% relative abundance in RC ecosystem. This phylum was largely dominated by the genus *Candidatus Nitrososphaera*, which responds positively to nitrogen fertilization and farmyard manure (Zhalnina *et al.*, 2013). This finding is associated with the lotic RC ecosystem in the agricultural catchment, where the QMST revealed a zoogenic origin of faecal water contamination (Table 1).

**Figure 1:**
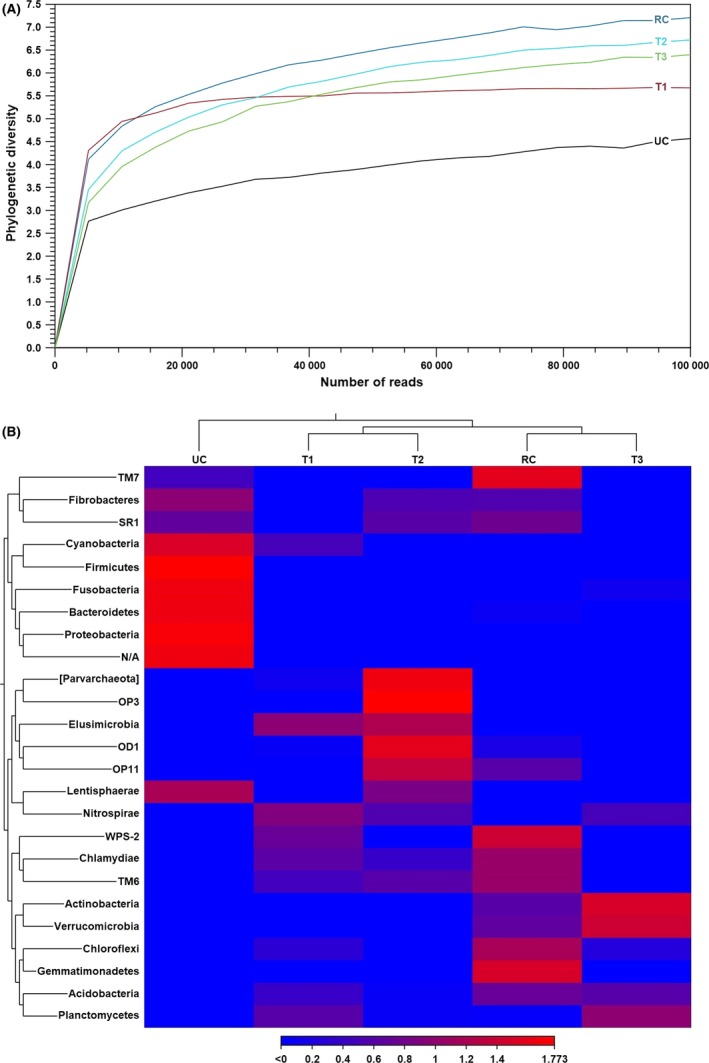
Diversity measures in the lotic ecosystems (a rural creek – RC, an urban creek – UC and three tributaries of different drinking water reservoirs – T1, T2 and T3). Phylogenetic alpha diversity (A) estimated from the rarefaction analysis expressing microbial species richness in each ecosystem. Beta diversity heat map (B) with hierarchical clustering of the ecosystems based on the microbial variety estimated through the Euclidean distance criterion. Both alpha and beta diversity were analysed using CLC Microbial Genomics Module version 2.5.1 (CLC Bio, QIAGEN Company, Aarhus, Denmark).

Rarefaction estimation as plotted in Fig. 1 discloses alpha diversity variations across the lotic ecosystems. The most diverse microbial composition was detected in the least faecally polluted ecosystem, while the highest decline of diversity was revealed in the extremely highly contaminated water, that is in ecosystems RC and UC respectively. These variations expose a remarkably negative impact of faecal pollution on aquatic microbial community structure. This can be related to various stress factors accompanying faecal water contamination that affect the basic ecological processes of ecosystems, leading to suppression of some microbes and enrichment of others, disruption in natural processes of biodegradation, water flow and nutrient availabilities. A negative response of the aquatic microbial community to the combined impacts of organic and inorganic pollutants, and variations in temperature, oxygen, salinity, acidification, nutrient and organic matter variabilities have been widely observed (Van Horn *et al.*, 2011; Pratt *et al.*, 2012; Mahmoudi *et al.*, 2015; Ibekwe *et al.*, 2016; Mesa *et al.*, 2017; Yao *et al.*, 2017).

A heat map of beta diversity (Fig. 1) illustrates the similarity/relatedness of the studied ecosystems in terms of their microbial community features. The performed hierarchical clustering was statistically significant (*P*‐value = 0.00097) as revealed by permutational multivariate analysis of variance (PERMANOVA). A group of two closely related clusters composed of ecosystems T1–T2 and RC–T3 was identified. Distinguishably, the UC ecosystem exhibited noticeable divergent microbial structure in comparison with the aforementioned group and stands as a separate cluster. This analysis clearly unveils that microbiota in the most faecally contaminated UC ecosystem, influenced mostly by the anthropogenic pollution sources (Table 1), was greatly deviated from the other ecosystems. Furthermore, a typical human gastrointestinal microbiome characterized mainly by *Proteobacteria*, *Firmicutes*, *Bacteroidetes*, *Synergistetes*, *Tenericutes* and *Fusobacteria* (David *et al.*, 2014; Goodrich *et al.*, 2014; Rowland *et al.*, 2018) was predominant in the UC ecosystem. This again correlates strongly with the outcomes of the QMST tests identifying the primarily human origin of faecal water contamination in this ecosystem (Table 1).

Both analytical and statistical tests validate the results of this study, which as a whole presents novel interpretations of the effect of anthropo‐zoogenic faecal pollution on aquatic microbial diversity in lotic waters. The outcomes clearly answer the research question addressed in this work by demonstrating a substantial reduction of the microbial diversity in water with severe faecal contamination. In addition, the study shows that the community compositions are distinct between ecosystems exposed to anthropogenic or zoogenic pollution. To conclude, the primary finding obtained in the current study is that the higher faecal water contamination the lower aquatic microbial diversity.

## Conflict of interest

None declared.
